# Evaluation of antimicrobial potential of leaf extract of plants collected in the UFAM campus' reserve

**DOI:** 10.1186/1753-6561-8-S4-P198

**Published:** 2014-10-01

**Authors:** Nani Carvalho, Ivens Siqueira, Raimundo Felipe Cruz Filho

**Affiliations:** 1 Universidade Federal Do Amazonas, UFAM, Manaus, AM, Brasil

## Background

The search for substances with antimicrobial properties is intensifying every day, given the increasing number of microorganisms resistant to the usual antibiotics. The use of plant components in the pharmaceutical field has gradually increased in Brazil. According to the World Health Organization, medicinal plants should be the best sources to obtain up new varieties of drugs [1]. Due to the above, there was the need to assess the antimicrobial potential in some species of Myrtaceae, Anacardiaceae and Oxalidaceae families.

## Methods

The leaves were collected in domestic and native plants in the reserve of the university campus. The collected material was triturated in distilled water, centrifuged 8000xg and the supernatant filtered through 0.22-µm membrane and stored in 15mL-Falcon tube [2]. The leaves were dried at 50 °C for 24h. The antimicrobial activity tests were the agar diffusion (Kirby-Bauer), and when confirmed the presence of antagonism one bioautography was performed to determine the Rf of antibiotic molecules. Test microorganisms were spiked in Plate Count Agar medium (PCA) and incubated at 37°C for 24 hours before testing [2]. For inoculum preparation, the youth cultures of each microorganism were standardized in sterile saline according to the 0.5-MacFarland Scale. The tested extracts included the filtrate liquid and the hydrated powder 10% (w/v). The seeding was carpet-type using "Swab", 100µL of extracts were placed in "Cup plate" incubated for 24 h at 37 °C. The positive extracts in the biological assays were weighed and serially diluted to twice with distilled water (200 mg / 2 mL) and transferring one mL of this dilution to the subsequent tube to 10^-8 ^in In each tube containing the respective dilution by adding 19.0 mL of Mueller-Hinton agar, which was poured into Petri dishes. Thus, the concentration range from 5 to 0.04 mg / mL was obtained. The seeding will be carpet type using "Swab". The MIC will be considered the lowest concentration that inhibited microbial growth [3].

## Results and discussion

Species of Myrtaceae, Anacardiaceae and Oxalidaceae famiies showed positive results against the test microorganisms *Candida albicans, Escherichia coli, Mycobacterium smegmatis *and *Staphylococcus aureus*. In the study concerning to the minimum inhibitory concentration (MIC), it was notorious that better results were obtained when using more concentrated extract. The mean inhibitory concentration for samples was 10^-²^.The results show that the species of the three families has great antimicrobial potential. Noting the ability of Amazon to research and production of new drugs.

## References

[B1] Bertini LM Pereira AF Oliveira CLL Menezes EA Morais SM Cunha FA Cavalcanti ESB Perfil de sensibilidade de bactérias frente a óleos e essenciais de algumas plantas do nordeste do Brasil Infarma 2005 17 3-4 80 83

[B2] Cunico MM Carvalho JLS Kerber VA Higaskino CEK Cruz Almeida SC Miguel MD Miguel OG Atividade antimicrobiana do extrato bruto etanólico de raízes e partes aéreas de OttoniamartianaMiq. (Piperaceae) Revista Brasileira de Farmacognosia 2004 14 2 97 103 10.1590/S0102-695X2004000200002

[B3] Fernandes TT Santos ATF Pimenta FC Atividade antimicrobiana das plantas: Plathymeniareticulata, Hymenaeacourbaril e Guazumaulmifolia Revista De Patologia Tropical 2005 34 2 113 122

